# Association of composite dietary antioxidant index with high risk of prostate cancer in middle-aged and elderly men: insights from NHANES

**DOI:** 10.3389/fimmu.2025.1530174

**Published:** 2025-02-18

**Authors:** Xuefeng Jin, Wenhui Tong, Li Sun, Sujue Lu, Pan Sun, Hangxu Li, Yan Liu

**Affiliations:** ^1^ Department of Urology, The First Affiliated Hospital of Jinzhou Medical University, Jinzhou Medical University, Jinzhou, Liaoning, China; ^2^ Medical College of Yangzhou University, Yangzhou, Jiangsu, China; ^3^ Medical College of Nantong University, Nantong, Jiangsu, China; ^4^ Medical College of Shaanxi University of Chinese Medicine, Xianyang, Shanxi, China; ^5^ Department of Urology, The Third Affiliated Hospital of Jinzhou Medical University, Jinzhou, Liaoning, China

**Keywords:** composite dietary antioxidant index (CDAI), dietary exogenous antioxidants, high risk for prostate cancer, NHANES, dietary therapy

## Abstract

**Objectives:**

In the US, the most common type of cancer and the second leading cause of cancer-related death in men is prostate cancer (PCa). Food and lifestyle factors may influence the risk of developing prostate cancer. Therefore, research on dietary components associated with prostate cancer is essential for its prevention. Data from the National Health and Nutrition Examination Survey (NHANES) between 2003 and 2010 was used for this cross-sectional investigation involving 5,658 middle-aged and older American men.

**Methods:**

Dietary antioxidant vitamins A, C, E, total carotenoids, zinc, and selenium were subtracted from the total mean, divided by the standard deviation, respectively, and then summed to become the CDAI. Participants were categorized as high risk for PCa if they had tPSA greater than 10 ng/mL or tPSA levels between 4 and 10 ng/mL with f/t PSA ratios of 25% or below; the remaining subjects were classified as being at low risk for PCa.

**Results:**

The sample represented approximately 75,984,602 American men. After multivariate logistic regression, dose-effect analysis and stratified analysis, CDAI was significantly and linearly negatively associated with a high risk of prostate cancer (OR=0.95, P=0.002, P for linear=0.0021). Age moderation analysis showed a significant effect on the inverse relationship between CDAI and prostate cancer risk (B = -0.0097, SE = 0.0034, t = -2.85, P = 0.004). Among the independent effects of CDAI components, zinc and selenium were more strongly negatively associated with prostate cancer (zinc, OR = 0.80, P = 0.008; selenium, OR = 0.78, P< 0.001).

**Conclusions:**

CDAI serves as a dietary indicator of prostate cancer risk in middle-aged and older men, and high dietary antioxidant intake has a significant protective effect on prostate cancer risk, especially in the older population of men.

## 1 Introduction

In the United States, prostate cancer (PCa) is the most common cancer type and ranks as the second leading cause of cancer-related death in men (1). In recent years, incidence and mortality rates have risen across age groups (2, 3), highlighting the necessity of investigating modifiable risk factors. Advances in genomic testing have enhanced our understanding of tumor aggressiveness and treatment strategies (4, 5), but increasing evidence suggests that modifiable lifestyle factors, such as diet, also play an important role in the prevention and management of prostate cancer. Therefore, due to the high cost and technical complexity of genetic testing, it may not be widely applicable in resource-limited settings. Dietary factors, as an accessible and cost-effective approach, help identify modifiable risk factors for prostate cancer, particularly in low-resource environments.

Systemic inflammation and immune response are believed to play key roles in tumor development and spread (6). Systemic inflammation is strongly linked to poor PCa outcomes, according to epidemiological studies (7, 8). As nutrition substantially influences chronic inflammation (9), pro-inflammatory diets have been linked globally to higher cancer risk and mortality (10, 11), with recent studies also connecting these diets to PCa (12, 13). However, the role of dietary antioxidants in PCa remains underexplored, particularly when considering composite dietary indices such as the Composite Dietary Antioxidant Index (CDAI), which aggregates the intake of various antioxidants. Most research in this area has focused on individual nutrients like vitamin E, vitamin C, and selenium, while there is limited evidence on how the collective intake of antioxidants affects PCa risk (14, 15). The Composite Dietary Antioxidant Index (CDAI), which measures a person’s dietary antioxidant capacity (TAC), is made up of antioxidants such as carotenoids, selenium, zinc, and vitamins A, E, and C (16).

Since 1994, prostate-specific antigen (PSA) has been used for PCa screening (17). While PSA testing facilitates early detection, limited specificity has raised concerns about overdiagnosis and overtreatment (18). PSA levels are influenced by a variety of factors, including not only prostate cancer (PCa) but also age, benign prostatic hyperplasia (BPH), prostatitis, and recent sexual activity (19). These factors can lead to false positives or false negatives, complicating the interpretation of PSA results. Furthermore, variations in PSA testing standards between different laboratories and methods may introduce additional variability, further affecting risk stratification. Therefore, PSA-based classification may not accurately reflect the true risk or aggressiveness of the disease. As a complementary tool, the f/t PSA ratio is particularly valuable when total PSA (tPSA) falls in the grey zone of 4-10 ng/mL, serving as a critical marker for further risk of cancer assessment (20). We examine the connection between CDAI and PCa risk by analyzing PSA data (f/t PSA ratios, fPSA, and tPSA) from NHANES (2003-2010).

## 2 Resources and procedures

### 2.1 Sources of information

NHANES is one of the National Center for Health Statistics’ primary initiatives (NCHS) (21). The program conducts a two-year survey cycle of a nationally representative sample of Americans who are not institutionalized (22). The NCHS Research Ethics Committee approved all procedures, and each subject provided informed consent (23). The cross-sectional study was deemed exempt from ethical review by the Academic Review Board due to the deidentified publicly available data used.

### 2.2 Study population

**Figure 1:**
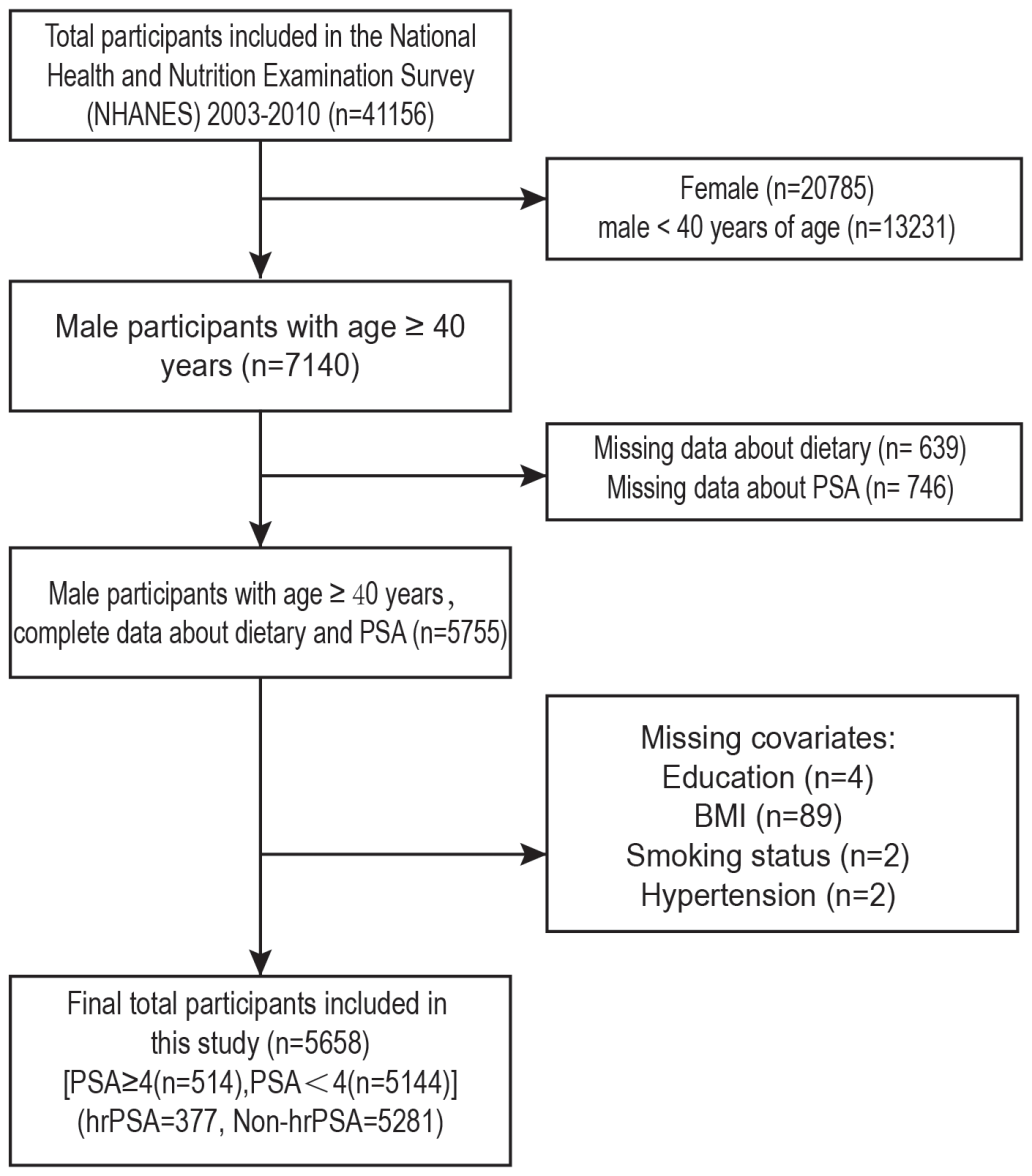
Research participant screening flowchart.

Data from four NHANES cycles (2003-2010) were analyzed, involving 41,156 participants (**Figure 1**). We excluded females (n=20,785) and males under 40 (n=13,231). Further exclusion criteria were: (1) Missing data about dietary (n=639); (2) missing PSA measurements (n=746); (3) missing education status (n=4); (4) unknown BM (n=89); (5) missing smoking status (n=2); (6) missing hypertension information (n=2). After the screening, 514 men with PSA ≥4 and the remainder with PSA<4 were included.

### 2.3 CDAI measurement

NHANES nutritional assessment includes a 24-hour dietary recall interview, conducted over two non-consecutive days to gather participants’ food intake data. Initial interviews were held at mobile examination centers (MECs) (24), and the subsequent interview occurred three to ten days later via phone (25). To minimize bias and improve reliability, the mean of the two measures was used. CDAI was calculated using a modified version of an existing model (26). Each micronutrient (vitamins E, A, C, selenium, zinc, and total carotenoids) was standardized by dividing by the standard deviation after subtracting the mean, which was then summed to derive the CDAI composite score. The formula is as follows:


$$CDAI=\sum_{i=1}^{n=6}(Individual\;Intake-Mean)/SD$$

### 2.4 Evaluation of PSA and risk for PCa

PSA testing was available to male participants aged ≥40 years who had no history of prostate cancer, prostate infection, inflammation, cystoscopy, rectal exam, or recent prostate biopsy. PCa risk was evaluated using the tPSA and f/t PSA ratios. Individuals with tPSA greater than 10 ng/mL, or tPSA between 4 and 10 ng/mL and an f/t PSA ratio of 25% or lower were categorized as high risk for PCa; others were considered low risk.

### 2.5 Covariate evaluation

Based on previous research findings, the following possible confounding variables were chosen for this study that may influence the risk of PCa: race, age, education, PIR, alcohol use, BMI, diabetes, hypertension, and total cholesterol levels (3, 27). Smoking is a known risk factor for various cancers, including prostate cancer, primarily through mechanisms such as increasing oxidative stress, DNA damage, and inflammation pathways. In the context of prostate cancer, smoking has been associated with more aggressive tumor types and poorer clinical prognosis (28). Regular physical activity has been shown to reduce the risk of prostate cancer, likely through mechanisms such as reducing systemic inflammation, improving immune function, and regulating hormone levels (e.g., testosterone). Physical activity also affects oxidative stress levels and overall health, which may, in turn, influence the role of dietary antioxidants (29). Therefore, considering these biological mechanisms, smoking and physical activity were included as covariates in the analysis to control for their potential confounding effects on the relationship between CDAI and prostate cancer.

We then grouped these covariates. Participants were categorized into two groups based on age (65 years):<65 years and ≥65 years. Educational attainment was divided into three categories: high school, higher education (above high school education for high education level), and lower education (below high school education for low education level). PIR< 2 was considered low, and PIR ≥ 2 was considered high. BMI was categorized into three groups: group 1 (<25), group 2 (≥25 and<30), and group 3 (>30). Participants were classified as smokers if they answered “yes” to smoking (SMQ020). In terms of alcohol consumption, the question about drinking (ALQ130) is used to distinguish between drinkers and non-drinkers. People who drink <12 drinks in a year are categorized as non-drinkers, while those who drink ≥12 drinks are categorized as drinkers.

Individuals with an average of three systolic blood pressure readings of ≥140 mmHg and diastolic blood pressure readings of ≥90 mmHg, those on antihypertensive medication, and those who responded “yes” to the question “Have you been told you have hypertension?” were considered patients with hypertension. Participants were considered to have diabetes if they answered “yes” to having diabetes, used glucose-lowering drugs or insulin, or had glycosylated hemoglobin (≥6.5%) and fasting blood glucose (≥126 mg/dl) values indicating diabetes. Total cholesterol levels were categorized as low (<240 mg/dl) or high (≥240 mg/dl).

### 2.6 Statistical analysis

During the processing phase, NHANES sample weights were applied to guarantee the study population’s national representation. Basic attributes were described after participants were classified into four CDAI categories. Weighted percentages (%) were used to compare categorical variables using a chi-square test. The results were displayed as mean (± SD) after weighted linear regression was used to compare continuous variables.

We first investigated the association between CDAI and prostate cancer risk in populations under various age groups due to the significant influence of age on prostate cancer risk (30, 31). We then explored the moderating effect of age on CDAI and prostate cancer risk. To ascertain whether a noteworthy trend or difference existed, the study performed univariate analyses and constructed three adjusted models to assess the connection between prostate cancer risk and CDAI. Model 1 was the baseline model, Model 2 added race, education, and PIR, and Model 3 further added BMI, diabetes, hypertension, smoking, alcohol consumption, cholesterol, and moderate and vigorous activity. Subsequently, the dose-response relationship was validated by GAM and threshold effect analysis was performed using RCS and smoothed curve fitting. CDAI and its components were separately regressed on prostate cancer risk to explore the relative effects. In addition, stratified associations between CDAI and UUI were explored by subgroup analyses, and interaction tests were performed.

For all statistical studies, R (version 4.4.0) was utilized. Statistical significance was established when the two-sided p-value was less than 0.05. Data were analyzed from November 1, 2023, to August 17, 2024.

## 3 Result

### 3.1 Population characteristics

The screening criteria led to the selection of 5658 eligible participants from NHANES 2003–2010 (**Figure 1**). Among them, 377 were at high risk for prostate cancer and 5281 were not. The weighted estimates for the baseline characteristics of the study population are shown in **Table 1**. Compared to those with lower levels of CDAI, those with higher levels of CDAI were more likely to be under 65 years of age, non-Hispanic, have an education level above high school, have a high PIR, be non-smokers, engage in vigorous physical activity, engage in moderate physical activity, not have hypertensive disease, and not have diabetes.

**Table 1:** Basic attributes of screened participants that are weighted (N=5658).

Characteristics	Total (n = 5658)	CDAI				P
		1 (n = 1415)	2 (n = 1414)	3 (n = 1414)	4 (n = 1415)	
**tPSA**	0.99 (0.58, 1.89)	1.05 (0.60,2.15)	1.00 (0.57,1.89)	1.00 (0.59,1.86)	0.90 (0.57,1.80)	**0.001**
**fPSA**	0.28 (0.18, 0.49)	0.30 (0.18,0.55)	0.29 (0.18,0.49)	0.29 (0.17,0.48)	0.27 (0.17,0.45)	**0.004**
**F/T**	0.29 (0.21, 0.38)	0.28 (0.21,0.38)	0.29 (0.22,0.38)	0.29 (0.22,0.38)	0.29 (0.21,0.38)	0.278
**hrPCa**						**0.003**
** No**	5281 (93.34)	1294 (91.45)	1315 (93.00)	1334 (94.34)	1338 (94.56)	
** Yes**	377 (6.66)	121 (8.55)	99 (7.00)	80 (5.66)	77 (5.44)	
**Age, years**						**<0.001**
** <65**	3607 (63.75)	801 (56.61)	860 (60.82)	921 (65.13)	1025 (72.44)	
** ≥65**	2051 (36.25)	614 (43.39)	554 (39.18)	493 (34.87)	390 (27.56)	
**Race**						**<0.001**
** Mexican American**	993 (17.55)	301 (21.27)	255 (18.03)	239 (16.90)	198 (13.99)	
** Other Hispanic**	370 (6.54)	113 (7.99)	88 (6.22)	90 (6.36)	79 (5.58)	
** Non-Hispanic white**	3067 (54.21)	611 (43.18)	782 (55.30)	810 (57.28)	864 (61.06)	
** Non-Hispanic black**	1031 (18.22)	344 (24.31)	241 (17.04)	219 (15.49)	227 (16.04)	
** Other**	197 (3.48)	46 (3.25)	48 (3.39)	56 (3.96)	47 (3.32)	
**Education**						**<0.001**
** Below high school level**	1736 (30.68)	647 (45.72)	457 (32.32)	371 (26.24)	261 (18.45)	
** High school diploma**	1337 (23.63)	330 (23.32)	346 (24.47)	337 (23.83)	324 (22.90)	
** More than high school**	2585 (45.69)	438 (30.95)	611 (43.21)	706 (49.93)	830 (58.66)	
**PIR**						**<0.001**
** <2**	2149 (40.72)	701 (54.17)	587 (44.34)	437 (33.26)	424 (31.52)	
** ≥2**	3128 (59.28)	593 (45.83)	737 (55.66)	877 (66.74)	921 (68.48)	
**Smoking**						**<0.001**
** Yes**	3498 (61.82)	950 (67.14)	888 (62.80)	863 (61.03)	797 (56.33)	
** No**	2160 (38.18)	465 (32.86)	526 (37.20)	551 (38.97)	618 (43.67)	
**Vigorous activity**						**<0.001**
** No**	4323 (76.41)	1178 (83.25)	1113 (78.71)	1041 (73.62)	991 (70.04)	
** Yes**	1335 (23.59)	237 (16.75)	301 (21.29)	373 (26.38)	424 (29.96)	
**Moderate activity**						**<0.001**
** No**	3166 (55.96)	943 (66.64)	804 (56.86)	741 (52.40)	678 (47.92)	
** Yes**	2492 (44.04)	472 (33.36)	610 (43.14)	673 (47.60)	737 (52.08)	
**Hypertension**						**<0.001**
** No**	2609 (46.11)	576 (40.71)	630 (44.55)	673 (47.60)	730 (51.59)	
** Yes**	3049 (53.89)	839 (59.29)	784 (55.45)	741 (52.40)	685 (48.41)	
**Diabetes**						**<0.001**
** No**	2088 (62.44)	489 (56.53)	513 (60.14)	546 (65.00)	540 (68.70)	
** Yes**	1256 (37.56)	376 (43.47)	340 (39.86)	294 (35.00)	246 (31.30)	
**Total cholesterol**						0.312
** Low level**	2003 (42.30)	473 (42.01)	485 (40.38)	518 (42.67)	527 (44.14)	
** High level**	2732 (57.70)	653 (57.99)	716 (59.62)	696 (57.33)	667 (55.86)	
**BMI (kg/m^2^)**						0.360
** <25**	1310 (23.15)	356 (25.16)	310 (21.92)	321 (22.70)	323 (22.83)	
** 25-29.99**	2386 (42.17)	584 (41.27)	589 (41.65)	615 (43.49)	598 (42.26)	
** ≥30**	1962 (34.68)	475 (33.57)	515 (36.42)	478 (33.80)	494 (34.91)	
**Alcohol consumption**						0.653
** No**	3546 (97.15)	758 (96.68)	877 (97.01)	925 (97.68)	986 (97.14)	
** Yes**	104 (2.85)	26 (3.32)	27 (2.99)	22 (2.32)	29 (2.86)	

Data are shown as n (%). OR, odds ratio; CI, confidence interval. All p-values less than 0.05 have been bolded.

Higher CDAI values were associated with lower levels of total PSA and free PSA, while the free-to-total PSA ratio was unaffected. The number of people at high risk for prostate cancer decreased significantly with higher CDAI values. In addition, we performed population-weighted analyses based on prostate cancer risk (**Supplementary Table S1**). The CDAI value for those at high risk for prostate cancer was -1.25 (-3.05, 0.82), which was significantly lower than the value of -0.58 (-2.49, 1.77) for those not at high risk (p=0.001).

### 3.2 Univariate analysis

The connection between prostate cancer and CDAI was initially explored by univariate analysis of total PSA and prostate cancer risk. High prostate cancer risk was positively associated with age ≥65 years, non-Hispanic black men, and hypertension. It was negatively associated with high school education or higher, BMI ≥25, and vigorous and moderate physical activity (**Supplementary Table S2**). Both total PSA (Q3, p=0.002; Q4, p<0.001) and high prostate cancer risk (Q3, p=0.004; Q4, p=0.001) were negatively associated with Q3 and Q4 of the CDAI quartiles.

### 3.3 Analysis of the moderating effect of age

**Figure 2:**
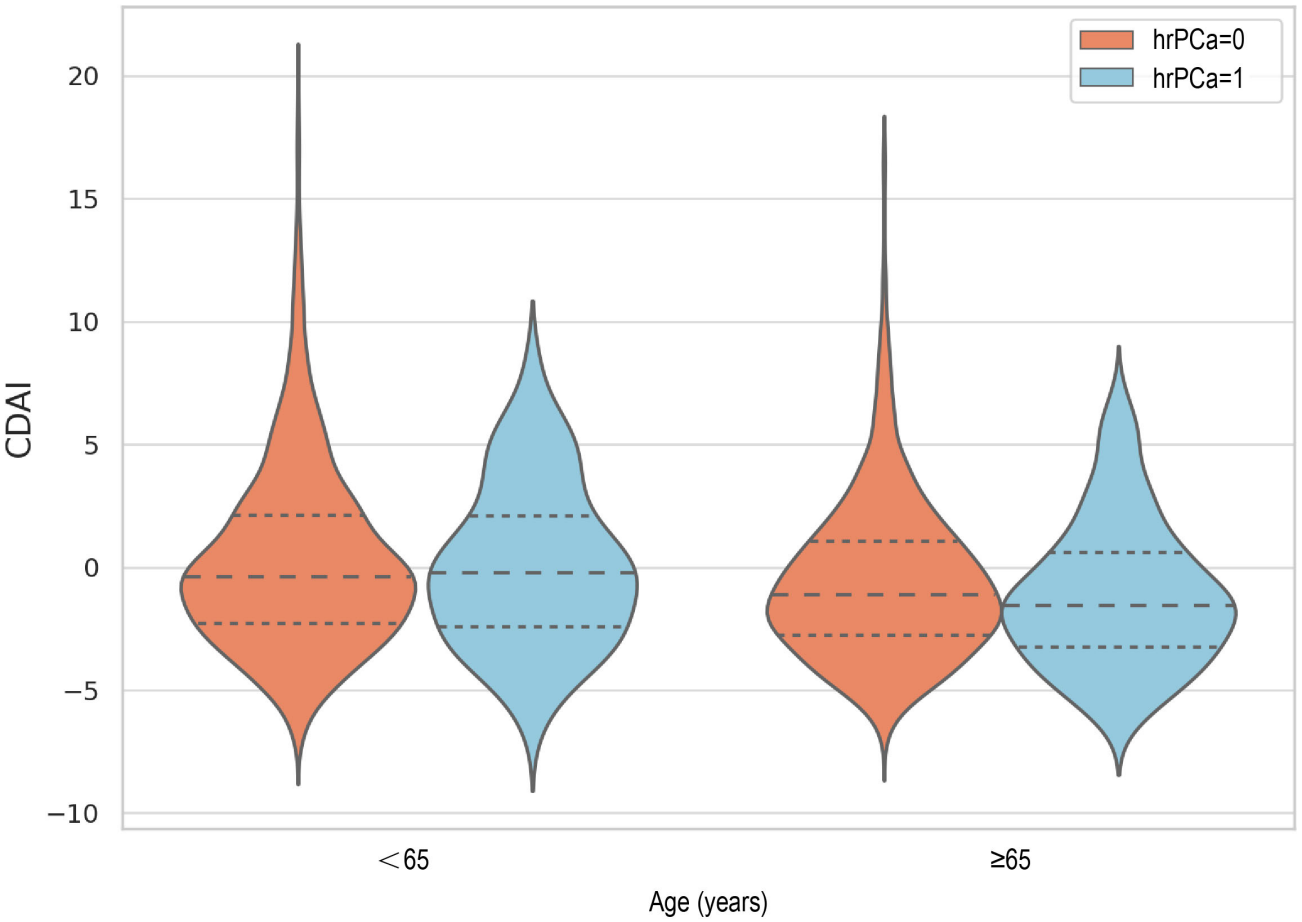
Violin plot of CDAl distribution (The dashed lines in the figure represent the quartiles. The medians from left to right are: -0.4, -0.2, -1.1, -1.5).

**Figure 3:**
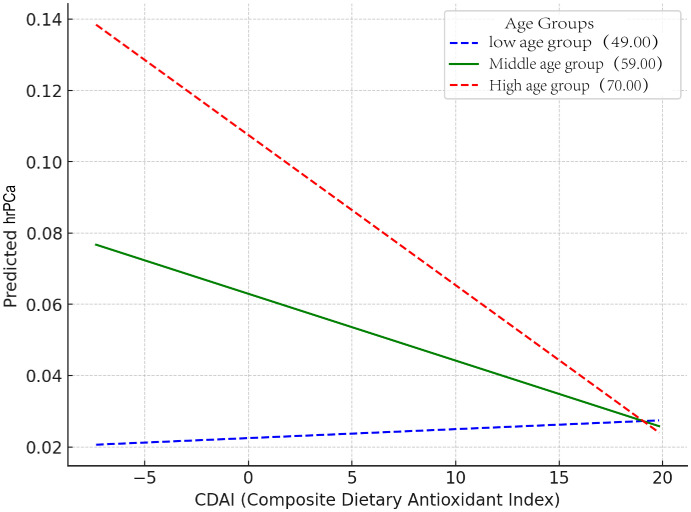
Interaction plot showing the relationship between CDAl and hrPCa in different age groups (The slopes for each group were: high age group: -0.0042, middle age group: -0.0019, and low age group: 0.0003).

The distribution of CDAI in relation to total PSA and prostate cancer risk in different age groups was analyzed and visualized as violin plots (**Figure 2**). In a moderated effects analysis with CDAI as the independent variable, hr-PCa as the dependent variable, and age as the moderator variable, the results indicated a significant moderating effect of age between CDAI and hr-PCa (B = -0.0097, P = 0.004). Visualized as an interaction plot showing the relationship between CDAI and hrPCa in different age groups (**Figure 3**). The findings demonstrated that the impact of CDAI on hr-PCa was enhanced with age, especially at the old age group (70 years), where the negative effect of CDAI was most significant. While in the younger age group (49 years), the effect of CDAI was weaker or even tended to be insignificant.

Subgroup analysis was conducted by dividing participants into four groups based on age: 40 ≤ age< 50, 50 ≤ age< 60, 60 ≤ age< 70, and 70 ≤ age (**Supplementary Table S2.1**). Overall Population: The intervention or exposure factor overall: reduced the risk of the outcome by approximately 7%, and the result was highly statistically significant. Age Subgroup: A significant effect was observed in the population aged 70 and above (OR: 0.94, P = 0.016), indicating that the intervention or exposure factor has a protective effect on the outcome in this group.

### 3.4 The relationships between CDAI and prostate cancer risk

**Figure 4:**
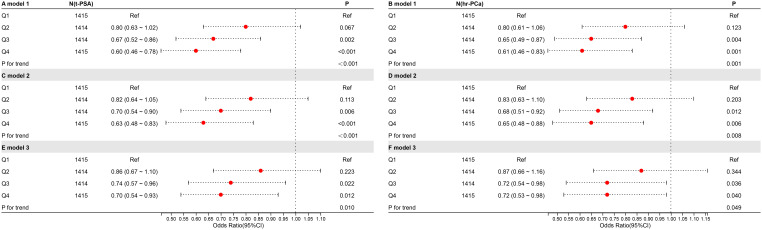
The relationship between CDAI and high risk of prostate cancer. The symbols “A,” “C,” and “E” represent t-PSA, whereas “B,” “D,” and “F” represent hr-PCa.

To further explore the association between CDAI and the high risk of prostate cancer, weighted logistic regression analyses were performed under three models (**Figure 4**). CDAI was converted to quartiles (Q1-4; Q1 was used as a reference). In Model One, no variables were added. In Model Two, PIR, race, and education were taken into account. Model Three was built on Model Two with adjustments for BMI, smoking, alcohol consumption, hypertension, diabetes, total cholesterol, moderate activity, and vigorous activity. In analyses with total PSA, Q3 and Q4 were negatively correlated in all three models. In model 3, OR=0.74, p=0.022 for Q3 and OR=0.70, p=0.012 for Q4. In the analysis of the prostate cancer risk, Q3 and Q4 were also negatively correlated in all three models. In model 3, OR=0.72, p=0.036 for Q3 and OR=0.72, p=0.040 for Q4.

### 3.5 Connectivity between CDAI and high risk of prostate cancer in terms of dose-response

**Figure 5:**
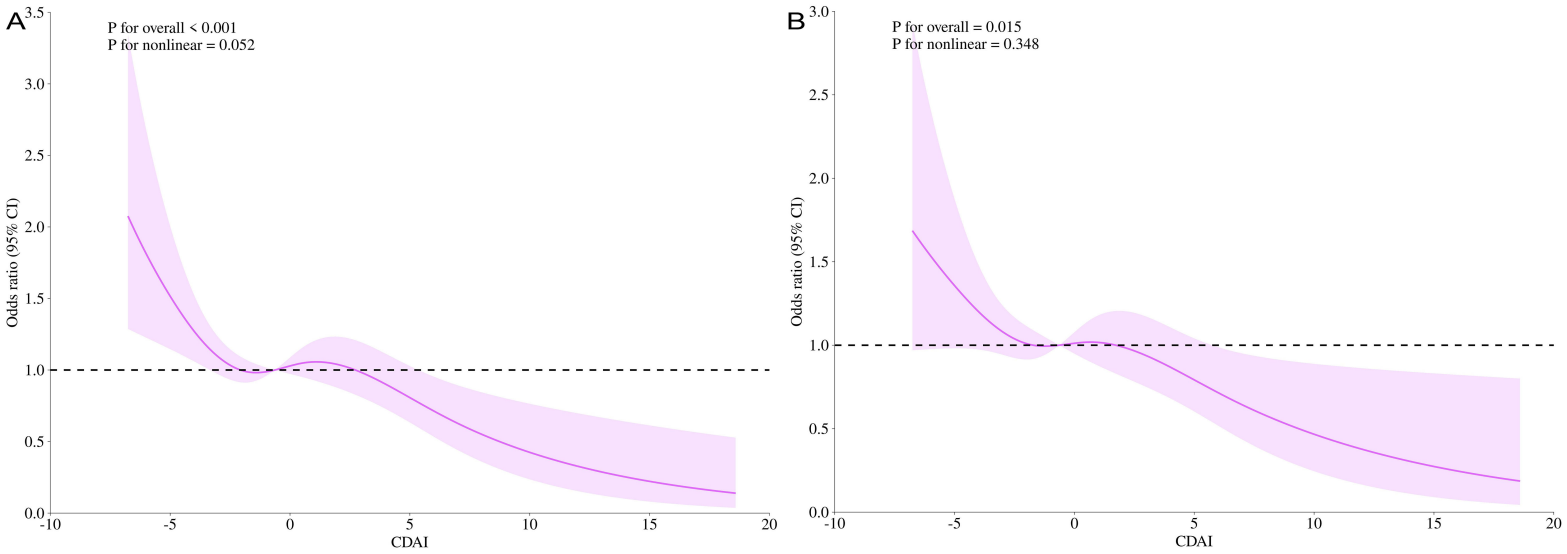
CDAI dose-response analysis and t-PSA/hr-PCa (RCS). t-PSA for the entire modified model **(A)**; hr-PCa for the entire modified model **(B)**. RCS, restricted cubic splines.

Under a fully adjusted model, the relationship between CDAI and high prostate cancer risk was investigated using smoothed curve fitting (**Supplementary Figure S1**). The RCS results for the dose-response relationship are displayed (**Figure 5**). Under the fully adjusted model, prostate cancer risk and total PSA were not nonlinearly correlated with CDAI (total PSA, p for nonlinear = 0.052, prostate cancer risk, p for nonlinear = 0.348). At low CDAI levels, the OR was greater than 1, suggesting a lower antioxidant index may be associated with higher health risk. The reference CDAI value at OR = 1 was -0.635, which can help guide men in avoiding prostate cancer. As the CDAI increased, the OR gradually decreased below 1, suggesting that a lower risk of prostate cancer is linked to a greater CDAI.

In addition, a threshold effect analysis between CDAI and prostate cancer risk was conducted (**Table 2**). In the model that has been entirely modified, the inflexion point (k) was 6.311 for t-PSA and 6.699 for hr-PCa (both log-likelihood ratios< 0.001). The linear model more fully explained the association between CDAI and high prostate cancer risk [OR = 0.943, P< 0.0001 (t-PSA); OR = 0.949, P = 0.0021 (hr-PCa)]. Furthermore, when CDAI > 6.699, the PCa risk was decreased by 58% for every additional CDAI unit (OR = 0.419, P = 0.0033).

**Table 2:** Relationship between CDAI and tPSA/hrPCa (Evaluation of the threshold impact).

Outcome	tPSA		hrPCa	
	OR (95% CI)	P	OR (95% CI)	P
Model one				
**Linear impact**	0.943 (0.916, 0.971)	**<0.0001**	0.949 (0.918, 0.981)	**0.0021**
Model two				
**Inflection point (K)**	6.311		6.699	
**CDAI< K**	0.973 (0.940, 1.007)	0.1232	0.980 (0.943, 1.020)	0.3230
**CDAI > K**	0.559 (0.362, 0.864)	**0.0088**	0.419 (0.188, 0.934)	**0.0333**
**Log likelihood ratio**	**<0.001**		**<0.001**	

The model is fully adjusted. All p-values less than 0.05 have been bolded.

### 3.6 Multiple regression analysis of the component-independent effects of the CDAI

To explore the connection between CDAI, each antioxidant ingredient, and PCa risk, a Pearson’s correlation analysis was conducted (**Supplementary Figure S2**). Z-scores were calculated for each of the six antioxidant components that make up the CDAI. There was a moderately high positive association between CDAI and the levels of each antioxidant, implying that CDAI better reflects overall antioxidant intake. Additionally, CDAI and prostate cancer risk were negatively correlated (r = -0.054).

**Figure 6:**
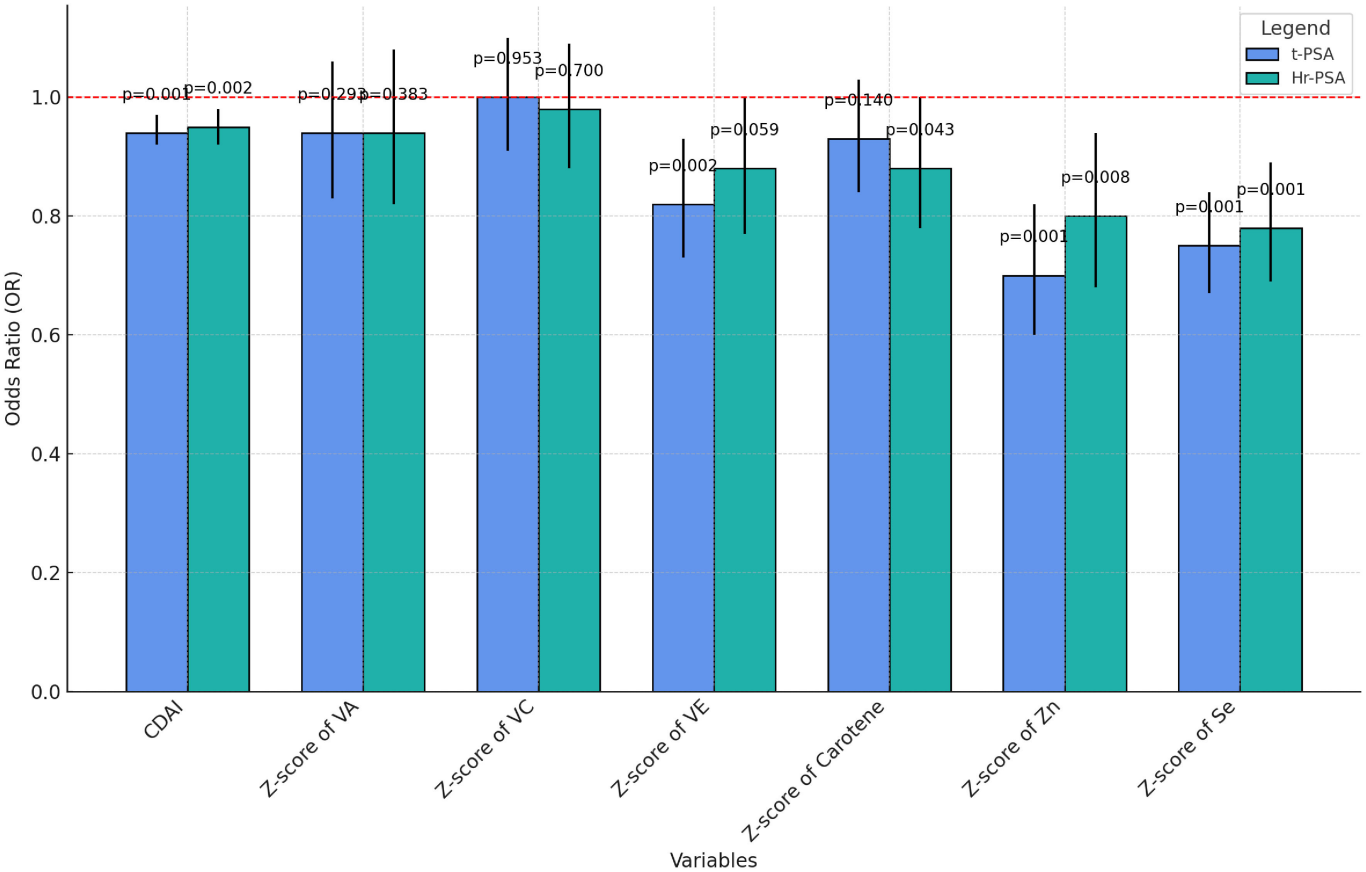
Grouped bar charts for regression analysis of CDAI and its components.

Regression analyses of CDAI and its components were performed (**Supplementary Table S3**) and visualized under a fully adjusted model (**Figure 6**). CDAI, zinc, and selenium showed significant negative correlations with t-PSA and hr-PCa. Vitamin A and C had no significant effect on prostate cancer risk, suggesting their role might be limited. Vitamin E was significantly negatively associated with total PSA and insignificantly negatively associated with a high risk of prostate adenocarcinoma. Total carotenoids were insignificantly negatively correlated with total PSA and weakly negatively correlated with hr-PCa (p=0.043).

### 3.7 Subgroup analysis between CDAI and high risk of prostate cancer

Stratified by age, race, PIR, education, hypertension, diabetes, BMI, smoking status, alcohol consumption, total cholesterol, and moderate- and vigorous-intensity exercise (**Table 3**), CDAI was significantly negatively correlated with both tPSA and hrPCa. In examining the relationship between CDAI and hr-PCa, an OR of 0.95 with p = 0.002 suggests that a decreased risk of PCa is linked to a greater CDAI.

**Table 3:** Stratified associations between tPSA/hrPCa and CDAl in adult males in the United States.

Subgroup	tPSA			hrPCa		
	OR (95%CI)	P	P for interaction	OR (95%CI)	P	P for interaction
All patients	0.94 (0.92 ~ 0.97)	**<0.001**		0.95 (0.92 ~ 0.98)	**0.002**	
Age (years)			0.082			0.255
<65	0.99 (0.95 ~ 1.04)	0.791		0.99 (0.94 ~ 1.04)	0.657	
≥65	0.94 (0.91 ~ 0.98)	**0.003**		0.95 (0.91 ~ 0.99)	**0.029**	
Race			0.898			0.292
Mexican American	0.93 (0.85 ~ 1.02)	0.124		0.94 (0.86 ~ 1.04)	0.247	
Other Hispanic	0.85 (0.74 ~ 0.97)	**0.016**		0.82 (0.70 ~ 0.96)	**0.013**	
Non-Hispanic white	0.94 (0.91 ~ 0.98)	**0.004**		0.95 (0.91 ~ 1.00)	**0.043**	
Non-Hispanic black	0.96 (0.90 ~ 1.02)	0.182		0.97 (0.91 ~ 1.04)	0.393	
Other	1.00 (0.80 ~ 1.25)	0.994		0.81 (0.60 ~ 1.09)	0.163	
Education			0.516			0.328
Below high school level	0.92 (0.87 ~ 0.97)	**0.003**		0.91 (0.85 ~ 0.97)	**0.005**	
High school diploma	0.97 (0.91 ~ 1.02)	0.251		0.97 (0.91 ~ 1.04)	0.366	
More than high school	0.94 (0.90 ~ 0.99)	**0.010**		0.96 (0.92 ~ 1.01)	0.099	
PIR			0.633			0.602
<2	0.95 (0.91 ~ 0.99)	**0.025**		0.94 (0.89 ~ 0.99)	**0.015**	
≥2	0.94 (0.90 ~ 0.97)	**0.001**		0.96 (0.92 ~ 1.00)	0.050	
BMI (kg/m2)			0.545			0.898
<25	0.96 (0.91 ~ 1.01)	0.128		0.95 (0.90 ~ 1.01)	0.133	
25-29.99	0.94 (0.90 ~ 0.98)	**0.008**		0.95 (0.90 ~ 1.00)	**0.044**	
≥30	0.93 (0.88 ~ 0.98)	**0.010**		0.94 (0.89 ~ 1.00)	0.062	
Smoking			0.648			0.948
Yes	0.94 (0.90 ~ 0.97)	**<0.001**		0.95 (0.91 ~ 0.99)	**0.022**	
No	0.95 (0.91 ~ 1.00)	**0.033**		0.94 (0.89 ~ 1.00)	**0.036**	
Vigorous activity status			0.246			0.240
No	0.94 (0.91 ~ 0.97)	**<0.001**		0.94 (0.91 ~ 0.98)	**0.002**	
Yes	0.97 (0.90 ~ 1.04)	0.326		0.98 (0.90 ~ 1.06)	0.584	
Moderate activity status			0.672			0.522
No	0.95 (0.91 ~ 0.98)	**0.005**		0.96 (0.92 ~ 1.00)	**0.033**	
Yes	0.94 (0.89 ~ 0.98)	**0.007**		0.94 (0.89 ~ 0.99)	**0.020**	
Hypertension			0.778			0.863
No	0.94 (0.89 ~ 0.99)	**0.016**		0.96 (0.91 ~ 1.01)	0.113	
Yes	0.94 (0.91 ~ 0.98)	**0.002**		0.94 (0.90 ~ 0.98)	**0.007**	
Diabetes			0.304			0.434
No	0.93 (0.90 ~ 0.97)	**<0.001**		0.94 (0.90 ~ 0.98)	**0.001**	
Yes	0.97 (0.91 ~ 1.03)	0.255		0.98 (0.91 ~ 1.05)	0.515	
Total cholesterol			0.477			0.481
Low level	0.93 (0.89 ~ 0.97)	**<0.001**		0.94 (0.89 ~ 0.98)	**0.009**	
High level	0.96 (0.92 ~ 1.00)	**0.032**		0.96 (0.92 ~ 1.01)	0.090	
Alcohol consumption			0.452			0.477
No	0.94 (0.91 ~ 0.97)	**<0.001**		0.95 (0.92 ~ 0.98)	**0.002**	
Yes	0.96 (0.00 ~ Inf)	1.000		0.96 (0.00 ~ Inf)	1.000	

Without altering the stratified variable itself, all covariates have been changed. OR, Odds Ratio; CI, Confidence Interval. All p-values less than 0.05 have been bolded.

The negative association between hrPCa and CDAI was more pronounced among those aged >65, other Hispanic, non-Hispanic white, with less than a high school education, low PIR, BMI between 25 - 30, no vigorous physical activity, hypertensive, without diabetes, with low cholesterol, and who did not consume alcohol. However, the interaction effect in both tPSA and hrPCa did not reach significance. Therefore, although a strong negative correlation was observed in some subgroups (e.g., low PIR, low education level, etc.), overall, the protective effect of CDAI was broadly applicable to different male populations.

## 4 Discussion

In this cross-sectional study using a nationally representative sample of U.S. men, a higher intake of antioxidant micronutrients was linked to a lower risk of prostate cancer (PCa). Specifically, the Composite Dietary Antioxidant Index (CDAI) was discovered to possess a noteworthy negative linear relationship with prostate cancer risk (p for linear = 0.002 in the threshold effects analysis; p for nonlinear = 0.348 in the RCS analysis). Moreover, age was found to significantly moderate the association between CDAI and PCa risk, with a stronger negative association observed in older age groups compared to younger ones. Another advantage of this study was the inclusion of physical activity, a significant influencing factor in PCa (27).

The part the systemic inflammatory response plays in PCa development and progression has been supported by various lines of evidence (32, 33). The SII (systemic immune-inflammation index), an indicator of the body’s inflammatory state, has shown a significant positive correlation with high prostate cancer risk (34, 35). However, the role of dietary antioxidant intake on PCa risk and systemic inflammation is under-explored. Our study was the first to examine the association between PCa risk and antioxidant dietary indices in older and middle-aged males who had never had PCa before.

Food is the source of most pro- and anti-inflammatory substances (36). Studies have found a higher correlation between inflammation and PCa risk, with higher levels of inflammatory biomarkers being positively associated with increased prostate cancer risk (37–42). There is also evidence linking prostate tumorigenesis to systemic and prostate inflammation (43–45). The inflammatory environment may promote cell growth in both benign and malignant prostate disorders (46). Immune cells in the tumor microenvironment (TME), such as neutrophils, platelets, and lymphocytes, have been shown to affect tumor growth and spread (47). By causing platelet aggregation in response to tumor cells, platelets can improve the potential for invasion of PCa stem-like cells, hastening the formation of PCa (48). By promoting aberrant angiogenesis, proangiogenic cytokines generated from platelets aid in the formation of PCa (49). The PCa-immunosuppressive TME has a sizable population of tumor-infiltrating neutrophils, which can encourage tumor growth and treatment resistance (50). Lower concentrations of some lymphocytes, namely CD4+ T cells and NK cells, have been demonstrated as being detrimental to patients with PCa and to increase treatment resistance (51).

**Figure 7:**
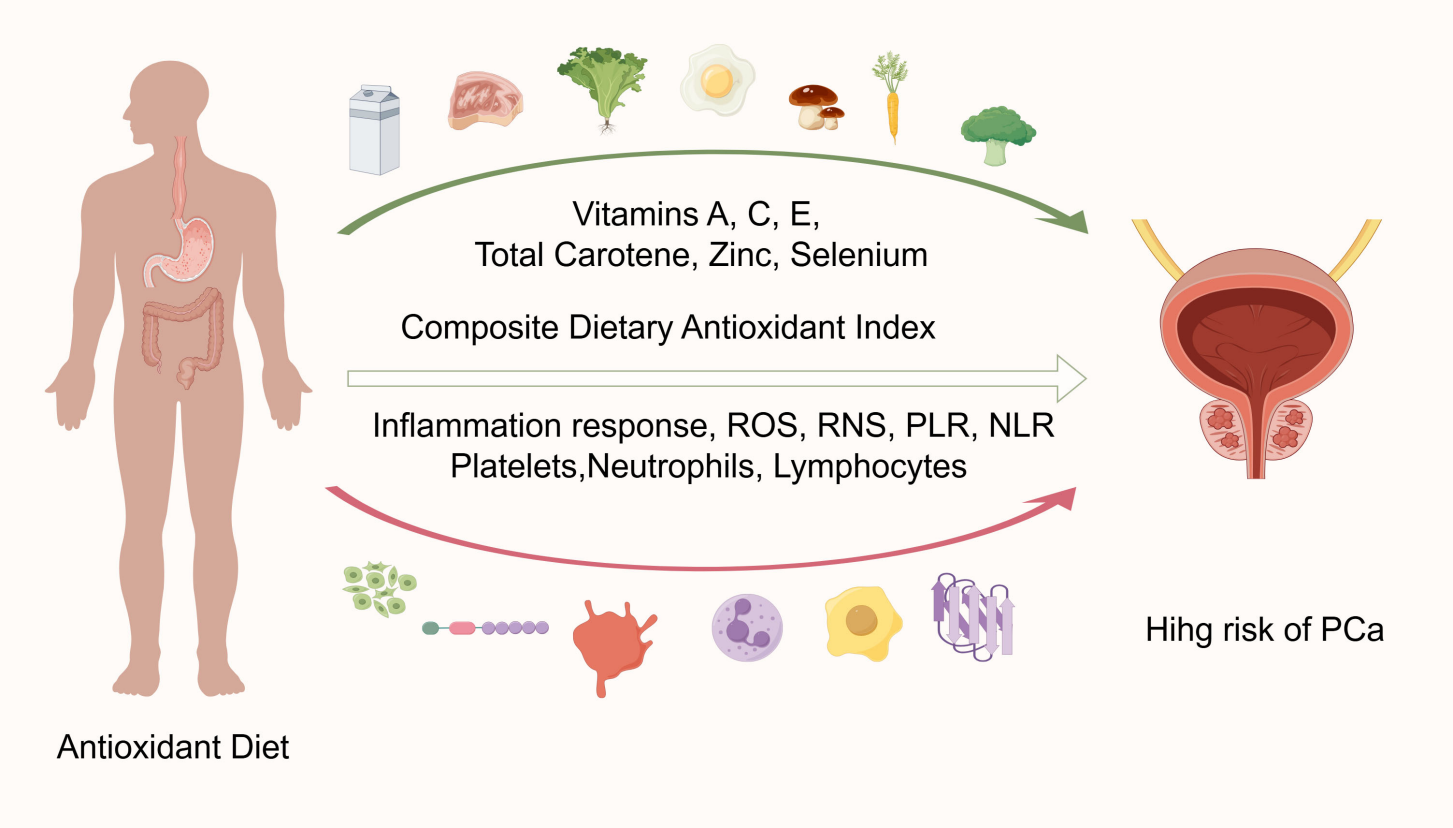
Visualization of CDAl and prostate cancer risk analysis (graphing via Figdraw). PLR, Platelet-lymphocyte ratio; NLR, Neutrophil-lymphocyte ratio.

Oxidative stress results from a mismatch between antioxidant and pro-oxidant production, which damages tissues and organs. Diet regulates the redox status of plasma and protects against environmental influences such as reactive oxygen and nitrogen species. Antioxidants scavenge oxidants to prevent oxidative damage and maintain a stable cellular redox state. A high CDAI level, indicating increased dietary antioxidant intake, led to a decreased inflammatory response both systemically and locally (52, 53). With lower PLR and NLR, middle-aged and elderly men who have never had PCa were associated with reduced PCa risk (**Figure 7**).

One established major risk factor for PCa is age (54). Prostate cancer growth is significantly positively correlated with advanced age, according to numerous research (55, 56). In our study, the moderating effect of age on the relationship between CDAI and prostate cancer risk was observed, with the negative correlation between CDAI and prostate cancer risk being more pronounced in older men. We believe this may be due to the lower dietary intake of older men compared to younger men, resulting in lower CDAI levels, which are positively associated with higher prostate cancer risk (57). In stratified analyses, interaction effects did not reach significant levels. Thus, although some subgroups (e.g., low PIR, low education level, etc.) showed strong negative correlations, the overall protective effect of CDAI was broadly applicable to a diverse male population.

Studies have shown that excess zinc inhibits prostate cancer growth, invasion, and migration (58, 59). According to a study by Eric A. Klein et al. published in JAMA, the risk of prostate cancer was inversely correlated with selenium intake, while vitamin E intake was positively associated (60). However, evaluating the total amount of antioxidants in the food could give a more comprehensive picture, considering the natural combinations of nutrients in food.

The strengths of this study include a comprehensive analysis of a nationally representative sample and careful consideration of multiple confounders, but there are some unavoidable limitations. First, since the study was cross-sectional, causality could not be established. Second, potential bias from interview-based dietary data may have led to biased results. Third, the database lacked testosterone data for participants from 2005-2010, making it unsuitable to include as a covariate. Moreover, the average daily consumption indicator among the covariates may not adequately capture drinking patterns, such as the distinction between binge drinking and sustained moderate drinking, thus limiting the ability to differentiate between these behaviors.

## 5 Conclusions

This study aimed to better understand the role of dietary antioxidants in the high risk of prostate cancer. The results showed a significant linear negative association between CDAI and prostate cancer risk, especially in older men.

## Data Availability

The original contributions presented in the study are included in the article/**Supplementary Material**. Further inquiries can be directed to the corresponding author.
