# Supplementary figures and images for: Association of composite dietary antioxidant index with high risk of prostate cancer in middle-aged and elderly men: insights from NHANES
Source: Front Immunol. 2025 Feb 18;16:1530174. doi: 10.3389/fimmu.2025.1530174 (PMC11876124; doi:10.3389/fimmu.2025.1530174)

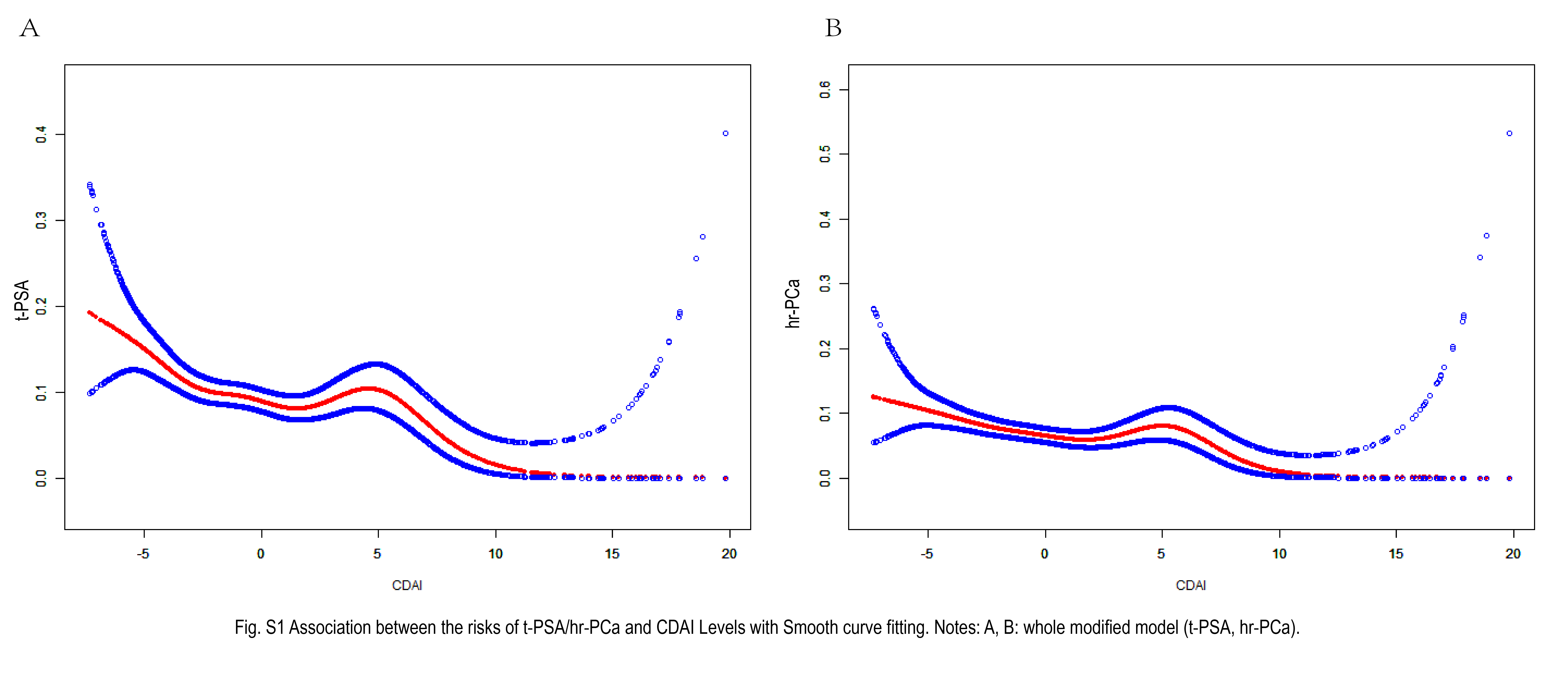

Supplement: Supplementary file 1 [file Image1.tif]

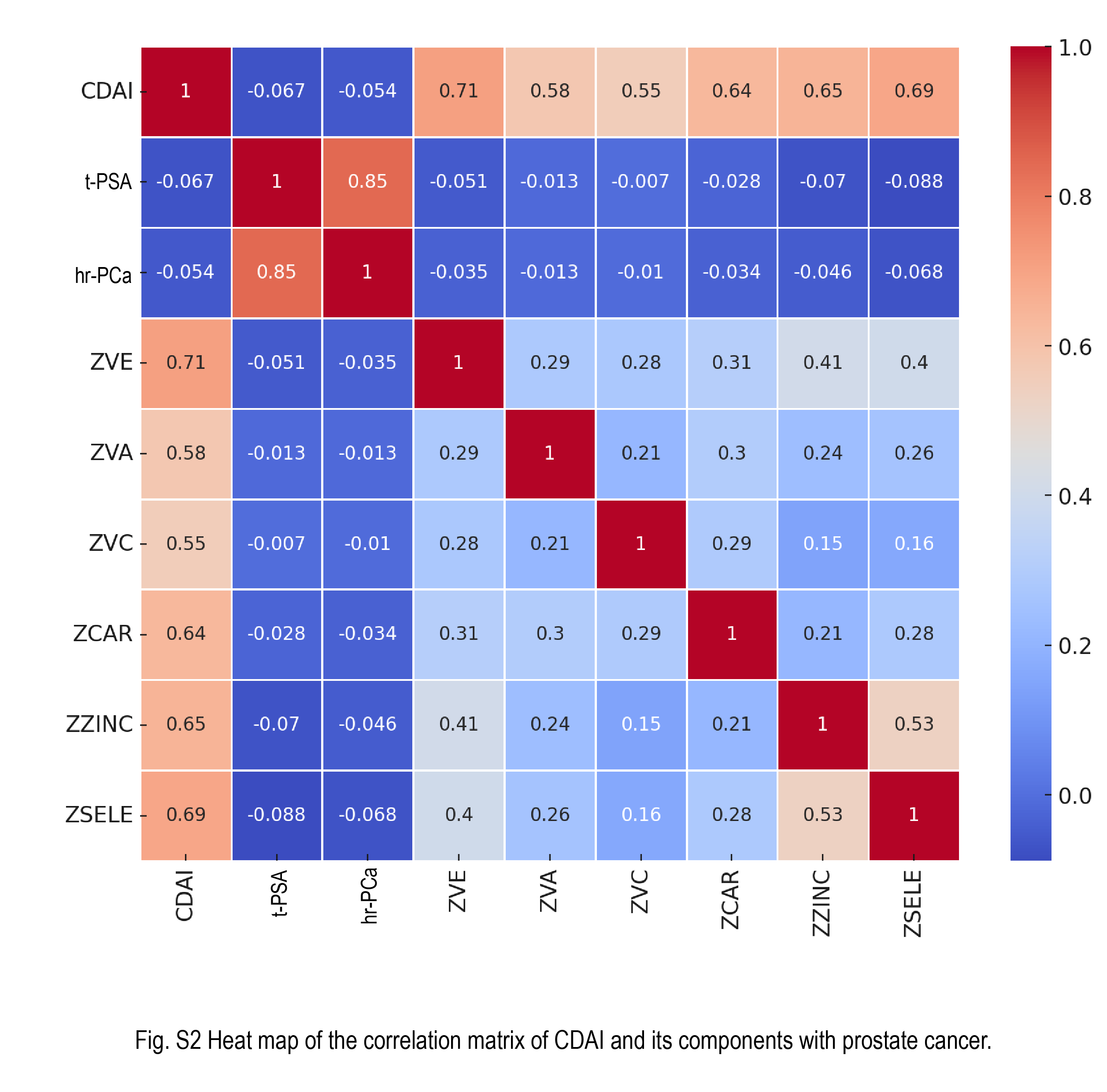

Supplement: Supplementary file 2 [file Image2.tif]
