# Supplementary material for: Association of composite dietary antioxidant index with high risk of prostate cancer in middle-aged and elderly men: insights from NHANES
Source: Front Immunol. 2025 Feb 18;16:1530174. doi: 10.3389/fimmu.2025.1530174 (PMC11876124; doi:10.3389/fimmu.2025.1530174)
Supplement: Supplementary file 3 [file Table1.docx]

Table S1. Weighted basic characteristics by prostate cancer high-risk classification

| Characteristics | Total (n = 5658) | High prostate cancer risk | | *P* |
| --- | --- | --- | --- | --- |
|  |  | No (n = 5281) | Yes (n = 377) |  |
| CDAI | -0.63 (-2.53, 1.73) | -0.58 (-2.49, 1.77) | -1.25 (-3.05, 0.82) | **<0.001** |
| Race |  |  |  | 0.106 |
| Mexican American | 993 (17.55) | 940 (17.80) | 53 (14.06) |  |
| Other Hispanic | 370 (6.54) | 345 (6.53) | 25 (6.63) |  |
| Non-Hispanic white | 3067 (54.21) | 2866 (54.27) | 201 (53.32) |  |
| Non-Hispanic black | 1031 (18.22) | 945 (17.89) | 86 (22.81) |  |
| Other | 197 (3.48) | 185 (3.50) | 12 (3.18) |  |
| Smoking |  |  |  | 0.906 |
| Yes | 3498 (61.82) | 3266 (61.84) | 232 (61.54) |  |
| No | 2160 (38.18) | 2015 (38.16) | 145 (38.46) |  |
| Vigorous activity |  |  |  | **<0.001** |
| No | 4323 (76.41) | 3996 (75.67) | 327 (86.74) |  |
| Yes | 1335 (23.59) | 1285 (24.33) | 50 (13.26) |  |
| Moderate activity |  |  |  | **0.002** |
| No | 3166 (55.96) | 2926 (55.41) | 240 (63.66) |  |
| Yes | 2492 (44.04) | 2355 (44.59) | 137 (36.34) |  |
| Hypertension |  |  |  | **<0.001** |
| No | 2609 (46.11) | 2476 (46.89) | 133 (35.28) |  |
| Yes | 3049 (53.89) | 2805 (53.11) | 244 (64.72) |  |
| Diabetes |  |  |  | 0.968 |
| No | 4402 (77.80) | 4109 (77.81) | 293 (77.72) |  |
| Yes | 1256 (22.20) | 1172 (22.19) | 84 (22.28) |  |
| Total cholesterol |  |  |  | 0.396 |
| Low level | 2926 (51.71) | 2739 (51.87) | 187 (49.60) |  |
| High level | 2732 (48.29) | 2542 (48.13) | 190 (50.40) |  |
| Education |  |  |  | 0.109 |
| Less than high school | 1736 (30.68) | 1603 (30.35) | 133 (35.28) |  |
| High school diploma | 1337 (23.63) | 1249 (23.65) | 88 (23.34) |  |
| More than high school | 2585 (45.69) | 2429 (46.00) | 156 (41.38) |  |
| PIR |  |  |  | 0.355 |
| <2 | 2149 (40.72) | 1997 (40.56) | 152 (43.06) |  |
| ≥2 | 3128 (59.28) | 2927 (59.44) | 201 (56.94) |  |
| BMI (kg/m^2^) |  |  |  | **0.006** |
| <25 | 1310 (23.15) | 1199 (22.70) | 111 (29.44) |  |
| 25-29.99 | 2386 (42.17) | 2231 (42.25) | 155 (41.11) |  |
| ≥30 | 1962 (34.68) | 1851 (35.05) | 111 (29.44) |  |
| Alcohol consumption |  |  |  | 0.081 |
| No | 3546 (97.15) | 3333 (97.03) | 213 (99.07) |  |
| Yes | 104 (2.85) | 102 (2.97) | 2 (0.93) |  |
| Age, years |  |  |  | **<0.001** |
| <65 | 3607 (63.75) | 3493 (66.14) | 114 (30.24) |  |
| ≥65 | 2051 (36.25) | 1788 (33.86) | 263 (69.76) |  |

Data are shown as n (%).
