# Supplementary material for: Association of composite dietary antioxidant index with high risk of prostate cancer in middle-aged and elderly men: insights from NHANES
Source: Front Immunol. 2025 Feb 18;16:1530174. doi: 10.3389/fimmu.2025.1530174 (PMC11876124; doi:10.3389/fimmu.2025.1530174)
Supplement: Supplementary file 4 [file Table2.docx]

Table S2. Univariate regression analysis

| **Variables** | **tPSA** | | **hrPCa** | |
| --- | --- | --- | --- | --- |
|  | OR (95%CI) | P | OR(95%CI) | P |
| CDAI Q4 |  |  |  |  |
| Q1 | 1.00 (Reference) |  | 1.00 (Reference) |  |
| Q2 | 0.80 (0.63 ~ 1.02) | 0.067 | 0.80 (0.61 ~ 1.06) | 0.123 |
| Q3 | 0.67 (0.52 ~ 0.86) | **0.002** | 0.65 (0.49 ~ 0.87) | **0.004** |
| Q4 | 0.60 (0.46 ~ 0.78) | **<0.001** | 0.61 (0.46 ~ 0.83) | **0.001** |
| Age (years) |  |  |  |  |
| **<65** | 1.00 (Reference) |  | 1.00 (Reference) |  |
| **≥65** | 5.96 (4.85 ~ 7.32) | **<0.001** | 4.51 (3.59 ~ 5.66) | **<0.001** |
| **Race** |  |  |  |  |
| **Mexican American** | 1.00 (Reference) |  | 1.00 (Reference) |  |
| **Other Hispanic** | 1.38 (0.89 ~ 2.13) | 0.152 | 1.29 (0.79 ~ 2.10) | 0.317 |
| **Non-Hispanic white** | 1.47 (1.11 ~ 1.94) | **0.007** | 1.24 (0.91 ~ 1.70) | 0.170 |
| **Non-Hispanic black** | 1.64 (1.19 ~ 2.26) | **0.002** | 1.61 (1.13 ~ 2.30) | **0.008** |
| **Other** | 1.33 (0.76 ~ 2.31) | 0.320 | 1.15 (0.60 ~ 2.20) | 0.671 |
| **Education** |  |  |  |  |
| **Below high school level** | 1.00 (Reference) |  | 1.00 (Reference) |  |
| **High school diploma** | 0.82 (0.64 ~ 1.04) | 0.106 | 0.85 (0.64 ~ 1.12) | 0.251 |
| **More than high school** | 0.74 (0.60 ~ 0.91) | **0.005** | 0.77 (0.61 ~ 0.98) | **0.036** |
| **PIR** |  |  |  |  |
| **<2** | 1.00 (Reference) |  | 1.00 (Reference) |  |
| **≥2** | 0.91 (0.76 ~ 1.09) | 0.299 | 0.92 (0.74 ~ 1.13) | 0.426 |
| **BMI (kg/m^2^)** |  |  |  |  |
| **<25** | 1.00 (Reference) |  | 1.00 (Reference) |  |
| **25-29.99** | 0.80 (0.64 ~ 0.99) | **0.049** | 0.75 (0.58 ~ 0.97) | **0.027** |
| **≥30** | 0.63 (0.49 ~ 0.80) | **<0.001** | 0.65 (0.49 ~ 0.85) | **0.002** |
| **Smoking** |  |  |  |  |
| **Yes** | 1.00 (Reference) |  | 1.00 (Reference) |  |
| **No** | 1.06 (0.88 ~ 1.28) | 0.519 | 1.01 (0.82 ~ 1.26) | 0.906 |
| **Alcohol consumption** |  |  |  |  |
| **No** | 1.00 (Reference) |  | 1.00 (Reference) |  |
| **Yes** | 0.19 (0.05 ~ 0.78) | **0.022** | 0.27 (0.07 ~ 1.10) | 0.068 |
| **Vigorous activity** |  |  |  |  |
| **No** | 1.00 (Reference) |  | 1.00 (Reference) |  |
| **Yes** | 0.51 (0.39 ~ 0.66) | **<0.001** | 0.48 (0.35 ~ 0.64) | **<0.001** |
| **Moderate activity** |  |  |  |  |
| **No** | 1.00 (Reference) |  | 1.00 (Reference) |  |
| **Yes** | 0.78 (0.65 ~ 0.94) | **0.008** | 0.71 (0.57 ~ 0.88) | **0.002** |
| **Hypertension** |  |  |  |  |
| **No** | 1.00 (Reference) |  | 1.00 (Reference) |  |
| **Yes** | 1.97 (1.62 ~ 2.39) | **<0.001** | 1.62 (1.30 ~ 2.01) | **<0.001** |
| **Diabetes** |  |  |  |  |
| **No** | 1.00 (Reference) |  | 1.00 (Reference) |  |
| **Yes** | 1.14 (0.92 ~ 1.41) | 0.225 | 1.01 (0.78 ~ 1.29) | 0.968 |
| **Total cholesterol** |  |  |  |  |
| **Low level** | 1.00 (Reference) |  | 1.00 (Reference) |  |
| **High level** | 1.07 (0.89 ~ 1.28) | 0.470 | 1.09 (0.89 ~ 1.35) | 0.396 |

Abbreviations: OR, odds ratio; CI, confidence interval.
