# Supplementary material for: Association of composite dietary antioxidant index with high risk of prostate cancer in middle-aged and elderly men: insights from NHANES
Source: Front Immunol. 2025 Feb 18;16:1530174. doi: 10.3389/fimmu.2025.1530174 (PMC11876124; doi:10.3389/fimmu.2025.1530174)
Supplement: Supplementary file 5 [file Table3.docx]

Table S3. Full factor-adjusted regression analysis of CDAI and its components

|  | *t-PSA* | | *Hr-PSA* | |
| --- | --- | --- | --- | --- |
|  | OR (95%CI) | *P* | OR (95%CI) | *P* |
| CDAI | 0.94 (0.92 ~ 0.97) | **<0.001** | 0.95 (0.92 ~ 0.98) | **0.002** |
| Z-score of VA | 0.94 (0.83 ~ 1.06) | 0.293 | 0.94 (0.82 ~ 1.08) | 0.383 |
| Z-score of VC | 1.00 (0.91 ~ 1.10) | 0.953 | 0.98 (0.88 ~ 1.09) | 0.700 |
| Z-score of VE | 0.82 (0.73 ~ 0.93) | **0.002** | 0.88 (0.77 ~ 1.00) | 0.059 |
| Z-score of Carotene | 0.93 (0.84 ~ 1.03) | 0.140 | 0.88 (0.78 ~ 0.99) | **0.043** |
| Z-score of Zn | 0.70 (0.60 ~ 0.82) | **<0.001** | 0.80 (0.68 ~ 0.94) | **0.008** |
| Z-score of Se | 0.75 (0.67 ~ 0.84) | **<0.001** | 0.78 (0.69 ~ 0.89) | **<0.001** |
