# Supplementary material for: Association of composite dietary antioxidant index with high risk of prostate cancer in middle-aged and elderly men: insights from NHANES
Source: Front Immunol. 2025 Feb 18;16:1530174. doi: 10.3389/fimmu.2025.1530174 (PMC11876124; doi:10.3389/fimmu.2025.1530174)
Supplement: Supplementary file 7 [file Table5.docx]

| Variables | n (%) | OR (95%CI) | *P* |
| --- | --- | --- | --- |
| All patients | 5658 (100.00) | 0.93 (0.90 ~ 0.96) | **<.001** |
| Age (years) |  |  |  |
| 40 ≤ age < 50 | 1526 (26.97) | 1.03 (0.91 ~ 1.16) | 0.617 |
| 50 ≤ age < 60 | 1343 (23.74) | 1.03 (0.95 ~ 1.11) | 0.503 |
| 60 ≤ age < 70 | 1352 (23.90) | 0.96 (0.91 ~ 1.01) | 0.152 |
| 70 ≤ age | 1437 (25.40) | 0.94 (0.89 ~ 0.99) | **0.016** |

Table S2.1 Age-based subgroup analysis was conducted under full adjustment for confounding factors.
